# CO_2_ Adsorption Capabilities of Functionalized Dyn[4]arenes

**DOI:** 10.1021/acsomega.5c08612

**Published:** 2026-03-17

**Authors:** Konstantinos Alektoridis, Eva Rauls

**Affiliations:** 198670 IMF, Universitetet i Stavanger Teknisk Naturvitenskaplege Fakultet, Stavanger, Rogaland 4021, Norway

## Abstract

Carbon dioxide (CO_2_), a natural gas present in the Earth’s atmosphere, continues to produce a greenhouse effect that locks our planet into a trajectory of warming. In the global effort to combat this concerning effect, various suggestions and ideas are constantly being considered and investigated. This report examines the CO_2_ adsorption capacity of two different dyn[4]­arene (D[4]­A) macrocycles by utilizing DFTB calculations. The most effective physisorption of the CO_2_ molecules has been found to take place inside the cavities for both structures. Furthermore, the ability of D[4]­A to capture multiple CO_2_ molecules until saturation is achieved has been investigated. Our findings highlight the unique properties of such macromolecules in adsorbing CO_2_ and indicate promising results for the A4 structures, in particular.

## Graphical abstract

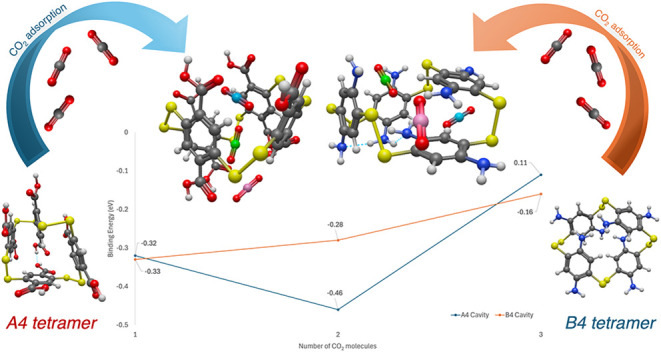

## 1 Introduction

As of February 2021, 124 countries have pledged to achieve carbon neutrality by 2050 or 2060, with the aim of reaching carbon neutrality by 2050 being to sustain global warming level to 1.5–2 °C above preindustrial levels.^1^ However, as the world’s population continues to grow, increasing demands in energy and food production lead to deforestation and heavy reliance on petroleum resources,^2^ which in turn lead to ever-increasing concentrations, due to anthropogenic activities,^3^ of global greenhouse gases (GHGs). These global emissions of GHGs are directly responsible for climate change, with CO_2_ remaining the largest contributor to global temperature rise.^4^

It becomes evident that by reducing CO_2_ emissions, carbon neutrality will become truly a feasible goal. Most of the reductions in CO_2_ come from technologies already in use,^1,2^ with carbon capture and sequestration (CCS) techniques gaining more research focus as time progresses,^5^ mainly due to their efficiency in decarbonization and technological viability.^6,7^ To reach net-zero emissions, it is imperative to explore new ways and ideas for capturing CO_2_ for its sustainable usage and storage, and for this reason many materials, such as metal oxides,^5,8^ metal organic frameworks (MOFs),^8,9^ zeolites,^10^ alloys,^11^ and soft organic frameworks (SOFs),^12,13^ among others, have been studied for their usage in CCS procedures. In particular, SOF materials have demonstrated promising results in CO_2_ capture due to their high selectivity (in the form of cavities) and framework flexibility, both allowing CO_2_ molecules to adsorb effectively,^13,14^ while also being chemically tunable (via the modification of the macrocycles),^15^ and thus have garnered a lot of attention from the industry.

Dyn­[*n*]­arenes are the dynamic analogues of pillararenes, with the methylene bridges between the benzene rings substituted by disulfide bridges in para positions.^16^ The first examples of such molecules were described in 2006 and later expanded by Leclaire et al. in 2016.^17,18^ Such *p*-cyclophanes are characterized by these −S–S– bonds, which connect *n* aromatic units (1,4-dithiophenols) forming macrocyclic structures with their walls creating a tubular cavity.^18^ Common sizes of the macrocycles are dyn[3] to dyn[7]­arenes, with the *n* = 4 and 5 variations being favored for their thermodynamic stability and better yields.^16,17,19^ D­[*n*]As possess the promising features needed for CO_2_ capture; more importantly, the cavity-like geometry and various binding sites in the form of functional groups. Furthermore, disulfides are prone to oligomerization under neutral pH in solution,^20^ allowing for the building blocks to self-assemble into dyn­[*n*]­arenes of various sizes, depending on the exploitation of the driving force.^16^

For this study, we chose to examine the A and B ortho-functionalized 1,4-diphenol units, each equipped with carboxyl (−COOH) and amino (−NH_2_) functional groups, respectively. The structures are listed in Figure 1.

**Figure 1:**
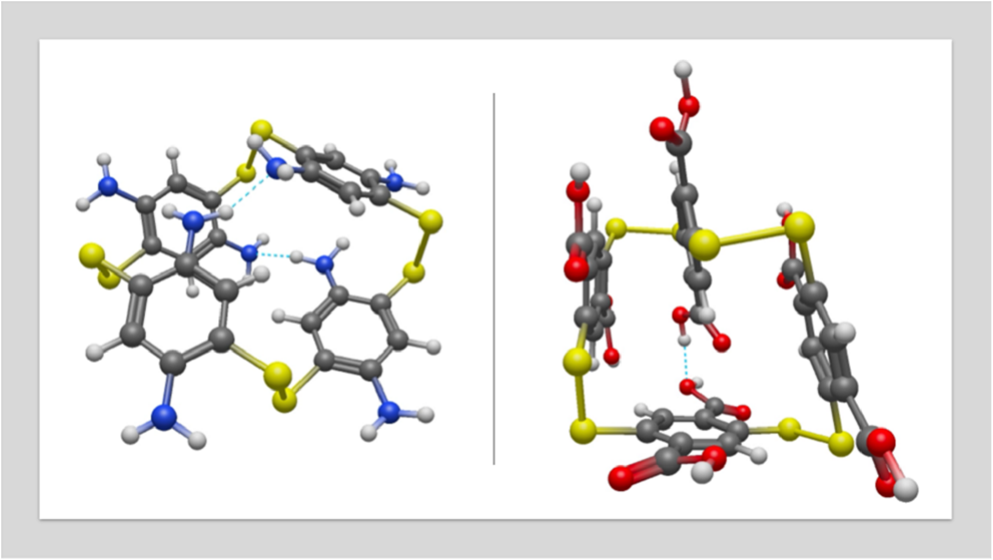
Perspective view for the optimized geometries of the B4 (left) and A4 (right) structures.

The monomer of 2,5-dimercaptoterephtalic acid (A) can be synthesized with commercially available reagents (such as diethyl 2,5-dihydroxyterephtalate) with up to a 95% yield.^16,17^ The dipole/quadrupole attraction between the carboxyl groups and CO_2_ molecules, combined with opportunities for hydrogen bonding, as well as the cavity formation that was recorded after relaxation of the initial unit, demonstrated a promising aspect for the capture of CO_2_ molecules.

The 2,5-diaminobenzene-1,4-dithiol unit (B) is commercially available, and although the amide couplings can be conducted with satisfying yields, side reactions have been observed during the process.^16,19^ The NH_2_ group can act as both a hydrogen-bond donor and acceptor.^16^

## 2 Computational Approach

We performed calculations with the self-consistent charge density-functional-based tight-binding (SCC-DFTB) method^21,22^ with the DFTB+24.1 code.^23,24^ For our calculations, the mio-1.1.0 basis set was employed.^21,25^ Such Slater–Koster files were used in particular because of their fitting nature with the experimental parameters, as the mio-1.1 set was developed for organic molecules that include O, N, C, H, and S. For the van der Waals correction scheme, the SimpleDftD3 dispersion block was incorporated in the DFTB+ code. The self-consistent electronic energy was set to converge to 10^–5^ eV. The maximum tolerance for the forces was set to 10^–4^ eV. The valence orbitals used were 2s, 2p, and 1s for the C, O, and N atoms and 1s for hydrogen. The validity of the modeling approach and comparability to ab initio results were investigated in our previous publications about similar molecular structures.^12,14,15^

## 3 Results and Discussion

The goal of this research endeavor was 3-fold: to examine the CO_2_ adsorption capabilities of the A and B dyn[4]­arene macromolecules, determine a viable number of CO_2_ molecules that can be adsorbed in the respective cavities of these structures, and understand the best registries for the adsorption of the CO_2_ molecules. Specifically, the examination of the dyn[4]­arenes’ potential for CO_2_ adsorption was influenced by research work, such as the study by Ho and Rauls on the adsorption of CO_2_ on pillar­[*n*]­arene structures^12,15^ and various other greenhouse gases,^14,26^ which demonstrated the potential of such macrocycles for the capture of small gases.

Hydrogen bonds and π–π interactions are the two main mechanisms for the physisorption of CO_2_ molecules in the A and B structures. This is in line with previous studies on macrocycles and aromatic hosts for CO_2_ capture,^27–29^ with the physisorption of CO_2_ in macrocyclic cavities being predominantly driven by hydrogen bonding to polar groups and π-π/dispersion interactions with the aromatic walls. The small sizes of the cavities of the optimized A4 and B4 structures, in combination with the effects of π-π interactions between CO_2_ and one or multiple benzene rings, lead to the favorable binding of CO_2_ molecules. The same effect has also been observed in the DFT study of pillar­[*n*]­arenes,^12^ where the π-π interactions between CO_2_ and the aromatic cavity walls of the macrocycles become stronger in smaller pillar­[*n*]­arenes. Concerning hydrogen bonding, this effect is possible via interaction of the CO_2_ molecules with the −NH_2_ and hydroxyls from the −COOH functional groups of B4 and A4 macrocycles, respectively.

The adsorption energy is calculated as

1 $$E_{\mathrm{ads}}=E_{D\left[4\right]A+CO_{2}}-\left(E_{D\left[4\right]A}+E_{CO_{2}}\right)$$

where *E*_ads_ is the binding energy of CO_2_ with the D[4]­A structures, with higher negative values indicating a stronger adsorption, *E*_D[4]A+CO_2__ is the total energy of D[4]­A with the adsorbed CO_2_ molecule, *E*_D[4]A_ is the total energy of the optimized macrocycle, and *E*_CO_2__ is the molecule energy of the isolated CO_2_ molecule. For the purposes of this investigation, various configurations of the CO_2_ molecule with D4A structures were tested. Among these, the three lowest energy structures (after optimization) were selected for both A4 and B4 and presented here. These were, namely, the configurations Cavity, Top, and Side, as demonstrated in Figures 2 and 3. The binding energies for these structures are reported in Table 1.

**Figure 2:**
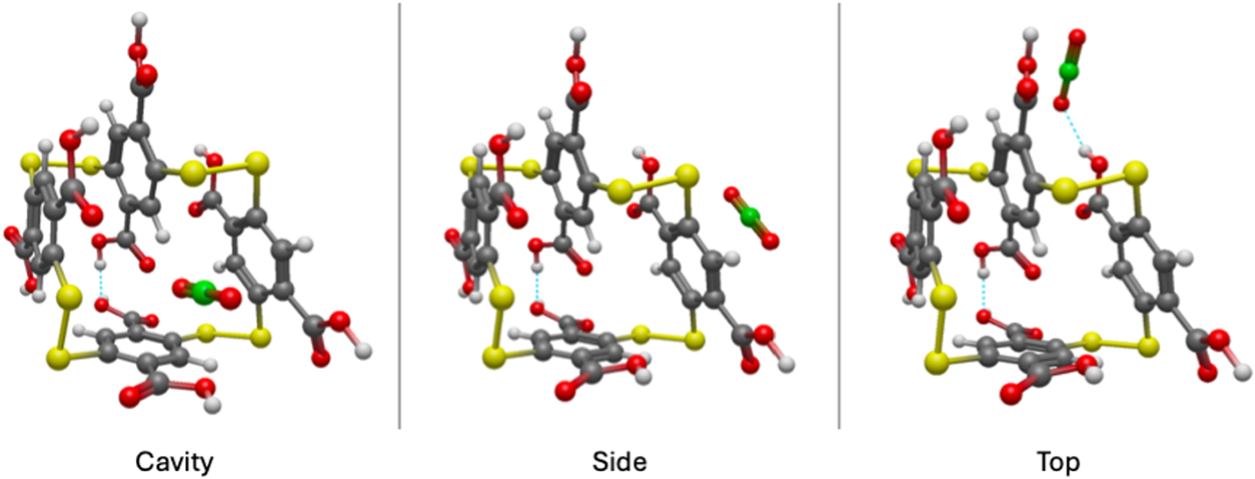
Perspective view of the Cavity, Side, and Top configurations of the A4 macrocycle after the adsorption of a single CO_2_ molecule. Hydrogen bonding is demonstrated with a blue dashed line.

**Figure 3:**
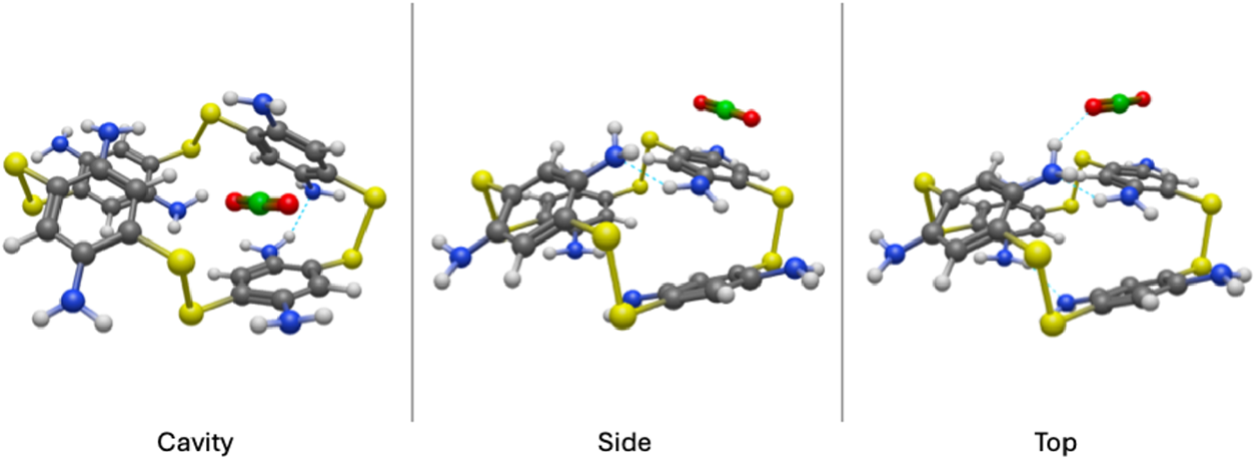
Perspective view of the three configurations of the B4 structure having successfully adsorbed 1 CO_2_ molecule. As with Figure 2, the blue dashed lines indicate the formation of hydrogen bonds.

**Table 1:** Various Configurations Used for the Positioning of a Single CO_2_ Molecule in and around the A4 Macrocycle^a^

	A4	B4
cavity	–0.32	–0.33
top	–0.21	–0.22
side	–0.15	–0.29

a All binding energies in eV.

For the calculation of the binding energy of each of the CO_2_ molecules that were progressively added into the D[4]­A structures, the following equation was used:

2 $$E_{\mathrm{ads}\_n}=E_{D\left[4\right]A+\left[n+1\right]CO_{2}}-\left(E_{D\left[4\right]A+nCO_{2}}+E_{CO_{2}}\right)$$

where the binding energy *E*_ads_*n*_ for each consecutively added CO_2_ molecule is demonstrated in Table 2. For this calculation, up to 3 CO_2_ molecules were progressively added. These molecules were placed within the D[4]­A structures with the best adsorption energies, i.e., the Cavity configurations for both A4 and B4 (cf. Table 1).

**Table 2:** Binding Energies (in [eV]) for Each of the CO_2_ Molecules for the D[4]­A Structures

A4 cavity	B4 cavity	# molecules
–0.32	–0.33	1 CO_2_
–0.46	–0.28	2 CO_2_
–0.11	–0.16	3 CO_2_

The results show the inability of the B4 structures to adsorb more than 1 CO_2_ molecule. This might be explained by their limited cavity size, leading to the rejection of further CO_2_ molecules by “pushing” them out of the cavity area due to space and π–π interactions between the benzene rings. This effect is less noticeable in the A4 macrocycles, in the cavity areas of which 2 CO_2_ molecules can be adsorbed. The role of their increased cavity sizes compared to B4 and the binding effects of the −COOH functional groups to CO_2_ molecules will be further investigated.

The binding energies of CO_2_ for the three selected configurations of A4 and B4 are presented in Table 1. The Cavity placement of the CO_2_ molecule was initially performed in the respective cavities of the structures, aiming for π–π interactions between the aromatic rings and the CO_2_ molecule. For the Top configuration, the CO_2_ molecule was placed near the functional groups that characterize each macrocycle with the purpose of achieving favorable hydrogen bonding sites. The Side configuration describes the interaction that occurs between D4As and the CO_2_ molecule when deposited near a benzene ring. For both functionalizations, the most preferable adsorption sites are found to be the Cavity structures of the macrocycles. During this study, it was noticed that the relaxed configuration of the aromatic rings leads eventually to a macrocycle with reduced cavity size, which can also be a feature that optimizes intra- and intermolecular bonds.

A simple analytical estimate for the calculation of the cavity area that the macrocycles possess before optimization is possible by considering the “squared” formation of the A and B tetramers. This way, the 2D area of the cavity can be calculated by measuring the average side length. Taking into consideration the geometric center of the macromolecules and using it as a reference point for the calculation of the cavity area, the length of the disulfide bonds can be used for examining the square or rectangular configuration of the structures before and after optimization.

For the calculation the following equation is used:

3 $$\mathrm{d̅}=\frac{1}{4}\sum_{i=1}^{4}d_{i}$$

where *d̅* is the average length for each side and *d*_*i*_ is the side length, calculated by measuring the distance between the four disulfide bond corner-points. The next step involves determining the formation of the initial D[4]­As. For the A4, the prerelaxed configuration had a roughly squared shape, while the optimized configuration had a more rectangular shape. The opposite was in effect for the B4 oligomer, with a rectangular early shape and a final square-like formation. For this reason, the following equations were used for the calculation of the cavity area:

4 $$A_{\mathrm{square}}={\mathrm{d̅}}^{2}$$

where *A*_square_ is determined to be the cavity area for a square configuration, and

5 $$A_{\mathrm{rectangular}}=d_{a}\times d_{b}$$

with *A*_rectangular_ being the cavity area for the rectangular formations and *d*_a_ and *d*_b_ being the average side length of the longer and shorter sides, respectively. By utilizing eqs 3, 4, and 5 the cavity areas of the A4 were calculated as 53.73 Å^2^ for the prerelaxed macromolecule and 48.65 Å^2^ for the optimized structure. The calculations for B4 were 51.83 and 48.30 Å^2^, for the same configurations.

The results indicate a shrinkage of the cavity area for both the A4 and B4 macrocycles, which occurred due to the geometrical optimization of the structures. Specifically, the cavity area decreased by ≈9.5% for A4 and ≈6.8% for B4.

The final binding energies for 1 CO_2_ molecule were calculated with eq 1. The most favorable configuration for CO_2_ adsorption in A4 is the Cavity, with a binding energy of −0.32 eV being the lowest energy of all of the other configurations. Similarly, the Cavity site of B4 demonstrated the lowest and strongest binding energy at −0.33 eV and is the best configuration for both D[4]­As. This preferable adsorption site can be explained due to the strong π–π interactions between CO_2_ and one or multiple aromatic rings, with the small size of the cavity enhancing the effect of this interaction, while the asymmetrical nature of the structures hinders the engagement of the CO_2_ molecule with all four benzene rings of the macrocycle.

The Top site configurations describe the attempts to bind the CO_2_ molecule with the hydroxyl group of the −COOH functional group of the A4 macrocycle with hydrogen bonding, in proximity and distanced accordingly, which indicated the binding of the CO_2_ molecule successfully through weak noncovalent bonds, such as hydrogen bonds or van der Waals forces. The same procedure was followed for the −NH_2_ group of B4.

The Side configurations of the CO_2_ molecules describe the various placements of the CO_2_ molecule in proximity of the D[4]­A structures’ aromatic rings, which lead to the observation of the weakest binding energy for the A4 and thus revealing that this site is not preferable for CO_2_ adsorption. However, the B4 Side yields a good binding energy of −0.29 eV. This can possibly indicate the preference of CO_2_ to bind with the benzene rings of the structure, perhaps for geometrical reasons. B4 exhibits an increased horizontal alignment of the benzene rings when compared to A4, where the more vertically placed rings deny such a binding site for the CO_2_ molecule.

In order to investigate the capacity of the D[4]­A macrocycles for CO_2_ uptake, we calculated binding energies of up to 3 CO_2_ molecules inserted into the Cavity sites of the A4 and B4 structures. These configurations were selected for both sites, as they had the best adsorption energies for a single CO_2_ molecule and would, thus, form the first step in multiple CO_2_ adsorption. The results are presented in Table 2 and Figure 6.

The calculation of the adsorption energies for 2 CO_2_ and 3 CO_2_ was performed with eq 2. A perspective view of the placement of the CO_2_ molecules is presented in Figures 4 and 5. While for the second CO_2_ molecule in the A4 structure, an increase of the adsorption energy to −0.46 eV was found, the insertion of the third molecule with the lowest binding energy of −0.11 eV did not lead to further energy gain upon CO_2_ adsorption.

**Figure 4:**
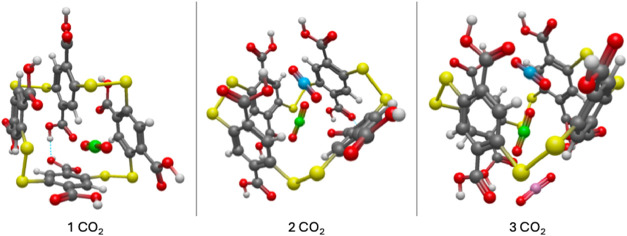
CO_2_ adsorption of 3 CO_2_ molecules in the Cavity configuration of A4, in perspective view. The first two molecules were adsorbed in the cavity of the structure, while the third is pushed out of the structure’s cavity. For better viewability, the first CO_2_ is depicted with a green colored C-atom, the second with cyan, and the third, with magenta.

**Figure 5:**
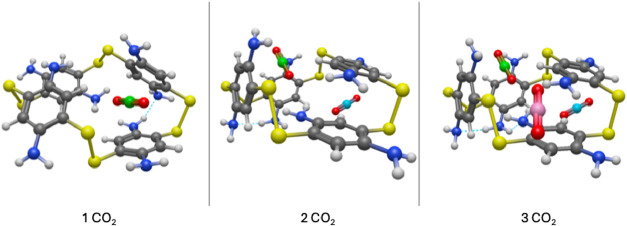
Perspective view of the B4 macrocycle with up to 3 CO_2_ molecules consecutively inserted. The first CO_2_ molecule is marked with green color, the second and third are noted with cyan and magenta, respectively.

In the B4 structure, the added second CO_2_ molecule is not effectively adsorbed, with −0.28 eV being a lower binding energy than that found for the first CO_2_ molecule.

In contrast to the A4 structure, the B4 structure appears to be unable to adsorb more than 1 CO_2_ molecule. Binding mechanisms, cavity size, and the −COOH functional groups of the A4 structure obviously allow for a more efficient CO_2_ uptake with an additional energy gain for a second CO_2_ molecule, while this is not the case for the NH_2_-functionalization of the B4 structure.

The dynarenes compare well with the related pillararene structures. The best adsorption energy of the D[4]­A structures has been found to be −0.33 eV, which is lower than the calculated CO_2_ binding energy for a pillar[4]­arene structure (−0.53 eV).^12^ However, only 1 CO_2_ molecule could be captured at the Cavity configuration of a P[4]­A, while the A4-functionalized D[4]­A can bind 2 molecules in its cavity. The larger and most commonly synthesized P[5]­A can bind 3 molecules within the cavity, and adsorption energies for each CO_2_ are in the same range as found for the D[4]­A.^12^ Different functionalizations of both macrocycles have to be studied for better efficiency and improved binding mechanisms.

The D[4]­A macrocycles have demonstrated the potential for efficient CO_2_ capture, as shown in Figure 6. Particularly, the A4 Cavity structure was observed to be able to capture up to 2 CO_2_ molecules. Further research directions of these systems include different functionalizations of D­[*n*]­A, different sizes, and more complex molecular aggregates. Different sizes of A­[*n*] and B­[*n*] macrocycles will affect and enhance the CO_2_ capture capability of the structures due to increased cavity sizes and more binding sites. Also, the disulfide-based macrocycles are modular and tunable,^20,30^ providing many adaptable binding sites.^17^ Self-organized surface adsorbates of the D­[*n*]­A structures are examined, focusing on the demobilization and stabilization of the macrocycles. This will, on the one hand, prevent deformation and cavity closure. On the other hand, additional intermolecular adsorption sites will be created on the surface, thus increasing CO_2_ capture capability. Furthermore, the adsorption selectivity of these systems will be studied by comparing the adsorption of other small gas molecules, especially polar molecules such as carbon or nitrogen monoxide or sulfur oxides.

**Figure 6:**
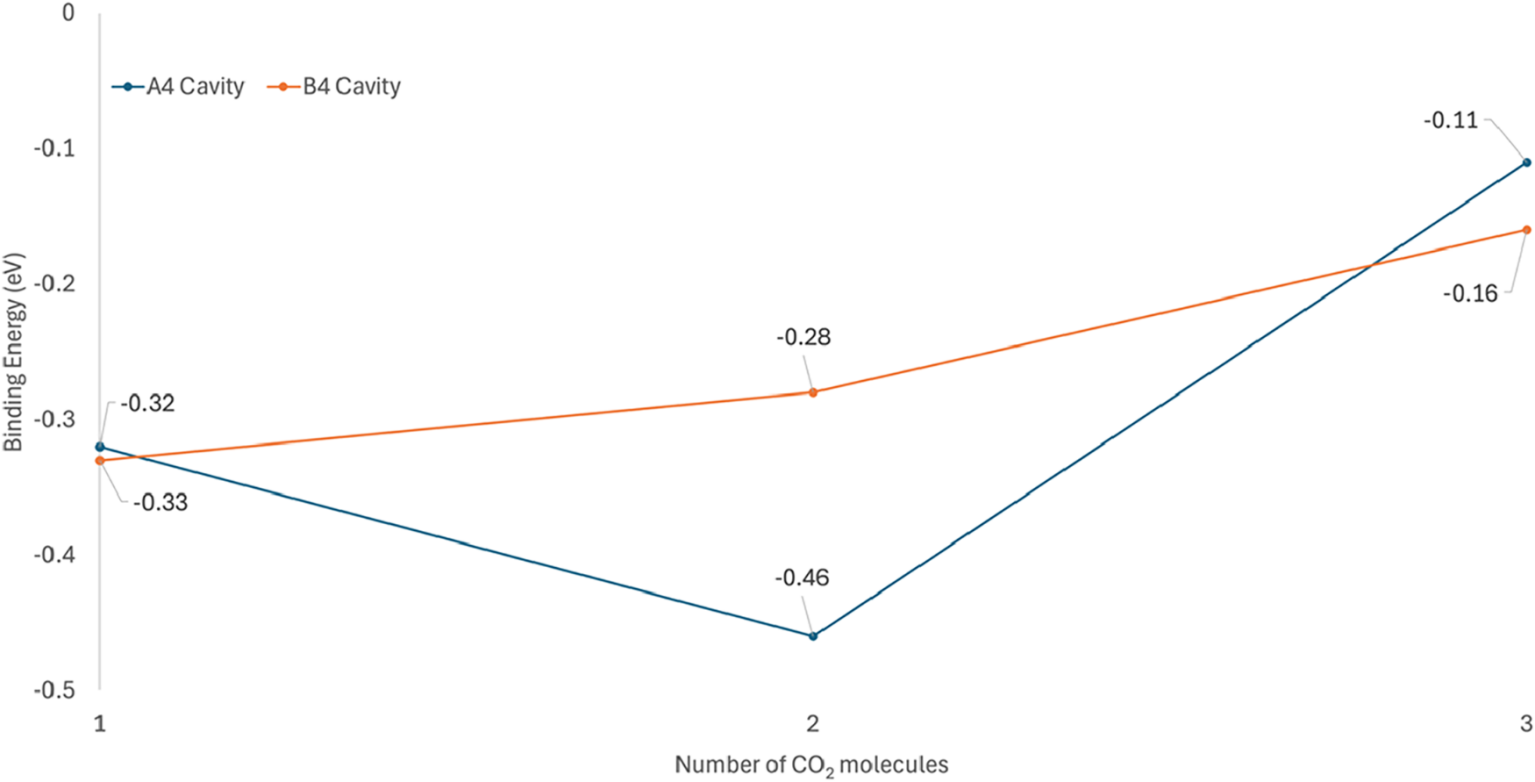
Schematic presentation of the adsorption energies in eV for the 3 CO_2_ molecules, for both A4 and B4 Cavity configurations. The A4 Cavity site is confirmed to be successful in adsorbing 2 CO_2_ molecules, with the transition to a lower binding energy of −0.46 from −0.33 eV.

Additional simulations were performed with −OH groups for the dyn[4]­arenes, therefore employing theoretical macromolecules such as 2,5-dimercapto-1,4-dihydroxybenzene or 2,5-dimercaptohydroquinone, dubbed (E), and used as building blocks for constructing the E4 tetramers. The subsequent calculations for CO_2_ adsorption initially exhibited a high binding energy (−0.32 eV) for 1 CO_2_ molecule in the E4 structures, binding strongly with the hydroxyls of the tetramer via a hydrogen bond. However, the limited cavity space of the E4 macromolecule proved to be a major hindrance to adsorbing effectively more than 1 CO_2_ molecules. Two more CO_2_ molecules interacted with the −OH groups of the structures, forming noncovalent hydrogen bonds, but neither was successfully adsorbed in E4 as the binding energies continuously rose for each consecutive CO_2_ molecule that was added.

One more candidate for examining its CO_2_ adsorption potential was the so-called C4 fluorine (–F) bearing macrocycle, with its monomers being 2,3,4,5-tetrafluorobenzene-1,4-dithiol units (C),^16^ for CO_2_ capture. Like the E4 structures, despite the successful adsorption of the first CO_2_ molecule (−0.28 eV), the C4 materials did not demonstrate good potential for binding more than 1 CO_2_ molecules. The main reason for this was the lack of a formed cavity area and the weak noncovalent interaction between the O atoms of the CO_2_ molecules and fluorine.

Finally, an evaluation of the supramolecular behavior of the macrocycles was investigated. For this purpose, several dimer (A4/A4 and B4/B4) configurations were optimized, including π-π stacked, sandwich-stacked, and hydrogen-bonded arrangements. The first configuration describes the interaction and stabilization of benzene rings in parallel arrangements at distances of ∼3.5–3.6 Å. The second configuration refers to the aggregation of the macrocycles in various registries, with the aromatic rings retaining their shifted form as occurred during the optimization of the individual D[4]­As. Lastly, the hydrogen-bonded arrangement provides a strong and highly directional interaction between the functional groups of the macrocycles.

Although π-π interactions generally contribute significantly to macrocycle association, particularly in planar aromatic systems where parallel-displaced stacking offsets Pauli repulsion while retaining dispersion forces,^31,32^ our calculations reveal that the hydrogen-bonded dimer for the A4 exhibits the most favorable aggregation energy (−1.69 eV), while ‘sandwich’-stacking the B4 macrocycles appears to be the best configuration for these dimers, with a total of −1.18 eV aggregation energy.

Hydrogen bonding provides a directional and highly specific stabilization mechanism, often exceeding the binding strength of dispersive stacking, particularly when donor/acceptor groups such as −COOH are present.^33,34^

Structurally, the optimized hydrogen-bonded A4/A4 dimer features H-bond lengths of approximately 1.7 Å, consistent with strong intermolecular hydrogen bonding.^35^ This optimized structure supports the interpretation that the most stable dimeric arrangement in this system occurs via hydrogen bonding, while π-π interactions contribute secondary stabilization in alternative (π-π stacked and ‘sandwich’-stacked) configurations.

For the B4/B4 dimer, the center-to-center distance of interacting benzene rings is 4.32 Å, and as the aromatic rings are not fully overlapping, this eventually leads to a larger centroid separation. It appears that the general structure of the macrocycles bearing the −NH_2_ functional groups is the main cause of pushing the individual D[4]As apart, creating a greater centroid distance, nonparallel orientation, and larger offsets. Since a cooperative network of noncovalent interactions, such as π-π and dispersion contributions, has been recorded in supramolecular assemblies,^36,37^ it can be argued that this is also the case for the aggregation of the B4 macrocycles.

## 4 Conclusion

Our results indicate the adsorption capabilities of two differently functionalized D[4]­A structures (A4 and B4). Both macrocycles demonstrate the highest binding energies for 1 CO_2_ molecule inside their cavities. The small cavity size of the tetramers and the optimized asymmetric formation lead to stronger π-π interaction between the benzene rings and CO_2_. For B4, a competing adsorption site is the Side configuration, in which π-π interaction between the benzene rings and the CO_2_ – outside the cavity – causes a binding energy slightly lower than that for the cavity adsorption site. Hydrogen bonding, as in the Top configurations, plays a minor role.

The A4 has been found to be able to bind a maximum of 2 CO_2_ molecules in its cavity site, while this is less likely for the B4. However, when looking past the Cavity configuration, the B4 macromolecule can also prove to be particularly useful with its characteristic −NH_2_ functional groups. During physisorption, the main interactions between an amino functional group and CO_2_ will lead either to the formation of a weak hydrogen bond, by providing polar, hydrogen-bonding sites that interact with the CO_2_ molecule, or in a dipole­(NH_2_)–quadrupole­(CO_2_) interaction. CO_2_ is a linear molecule (=C=O) with no permanent dipole but provides a significant quadrupole (partial negative charge on the oxygen atoms and partial positive charge on the carbon). Therefore, the B functionalization might be of particular interest when investigating surface-supported aggregates of the D­[*n*]­A with additional intermolecular adsorption sites. These investigations are under work and will be published elsewhere.
